# Evaluating How University Students Adapt to Stress: Psychometric Validation of a Psychological Instruments Battery

**DOI:** 10.3390/epidemiologia7020049

**Published:** 2026-04-03

**Authors:** Clara Simães, Catarina Morais, Liliana Fontes, Adérito Seixas, Rui Gomes

**Affiliations:** 1 School of Nursing, University of Minho, 4710-057 Braga, Portugal; csimaes@ese.uminho.pt; 2 Health Sciences Research Unit: Nursing (UICISA: E), University of Coimbra, 3004-531 Coimbra, Portugal; 3 Research Centre for Human Development, Faculty of Education and Psychology, Universidade Católica Portuguesa, 4169-005 Porto, Portugal; ctmorais@ucp.pt; 4 Adaptation, Performance, and Human Development Research Group, School of Psychology, University of Minho, 4710-057 Braga, Portugal; liliana.magalhaes2@gmail.com; 5 FP-I3ID, FP-BHS, Escola Superior de Saúde Fernando Pessoa, 4200-256 Porto, Portugal; aderito@ufp.edu.pt; 6 Psychology Research Centre, School of Psychology, University of Minho, 4710-057 Braga, Portugal

**Keywords:** academic stress, cognitive appraisal, coping, academic achievement expectations, higher education students

## Abstract

**Background**: In modern society, increased awareness of stress stems mainly from the pressures of competitive environments, where the pursuit of academic and professional success places substantial demands on individuals, who must adapt. Drawing on the Transactional Model and the Interactive Model of Human Adaptation to Stress, this paper presents a battery of instruments designed to comprehensively assess university students’ adaptation to stress. **Methods**: Data were collected from two academic years, using two independent samples of students: a calibration sample (*n* = 561) and a validation sample (*n* = 370) to test the psychometric properties of the instruments. The evaluation protocol included the Stress Questionnaire for Students (SQS), the Primary and Secondary Cognitive Appraisal Scale (PSCAS), the Reduced Coping Inventory (Coping-R), and the Academic Achievement Expectations (AAE). **Results**: Psychometric validation analyses indicated the best versions of the instruments’ battery. Namely, an 18-item version and a six-factor structure for the SQS, a 10-item version and a five-factor structure for the PSCAS, a 12-item version and a four-factor structure for the Coping-R, and a five-item, one-factor structure for the AAE. **Conclusions**: The proposed instruments can serve as a compound resource for screening for academic stress experiences in university students, and as an original tool to understand the entire process of stress adaptation.

## 1. Introduction

Stress, originally a concept rooted in physics, has become a complex and multidimensional phenomenon increasingly relevant across several fields, such as medicine, psychology, and education. Nowadays, increased awareness of stress is driven by the pressures of competitive societies, where the pursuit of academic and professional success imposes significant demands on individuals [1]. This change required reevaluating stress as both a biological response and as a reflection of how individuals interact with ever-changing educational and work environments [1,2]. In this framework, occupational stress is viewed as a societal concern and is considered by the World Health Organization as one of the most significant challenges of the twenty-first century, since research shows that stress levels have risen for decades, affecting individuals, families, organizations, and nations [3]. A similar scenario happens in higher education, where education can be a positive and transformative influence on students’ lives, but academic stress can and has become a serious and concerning problem [4,5]. In fact, the university context represents a critical stage in student development due to new academic responsibilities and demands and there is evidence that students are particularly vulnerable to stress [6].

Research indicates that students face several sources of stress, most notably those related to academic performance (tests/examinations) [7], the learning process [8], financial pressures [9], transition to university and overall expectations [10], work overload [11], and decreased motivation [12], among others. All these sources of stress can have adverse effects on students’ mental, emotional, and physical well-being, as well as their academic performance. Specifically, high levels of academic stress are strongly correlated with increased depression, anxiety, and other mental health problems [13,14,15], with some studies showing that a substantial portion of students experience sleep disturbances [14,15] and report symptoms such as difficulty concentrating, which in turn negatively impacts their academic performance [14]. Additionally, academic stress can lead students to adopt disruptive behaviors, such as alcohol and drug abuse [16], and experience physical symptoms, such as headaches and feelings of exhaustion, further impairing their ability to concentrate and maintain motivation [17], ultimately leading to decreased academic success and possible withdrawal [18,19]. Finally, stress often forms a negative feedback loop, in which declining academic performance worsens stress levels and vice versa, highlighting the need for early interventions and comprehensive support systems within the university environment [20]. Therefore, addressing academic stress is crucial for enhancing students’ overall well-being and academic success. In this study, the interactive and transactional perspective of adaptation to stress [21,22,23,24] was employed as a theoretical framework to propose a series of measures to understand academic stress among higher education students.

The interactive model of human adaptation to stress [22], based on Lazarus’s [21,24] transactional model, not only reinforces the importance of stressors in explaining how people adapt to work but also emphasizes the cognitive appraisal processes through which individuals respond to work conditions (i.e., how individuals evaluate their activities). The model indicates that stress phenomena and the individual’s adaptive outcome are explained by the interaction of stressors’ characteristics, cognitive appraisals, and reactions to stress, all influenced by personal and situational factors [22,25]. Thus, the model highlights six key aspects that explain how humans adapt to stressful situations. Specifically, the model considers the importance and features of the stressful event itself during the adaptation process, as well as the influence of antecedents and pre-existing factors—both situational (e.g., type of organization) and personal (e.g., personality traits)—on how individuals respond to stress. The central role of cognitive appraisal, highlighted as crucial in how humans adapt to stressful events, involves an individual’s interpretation and evaluation of the situation (i.e., primary cognitive appraisal), their coping resources, and control over the stressful event (i.e., secondary cognitive appraisal). Primary cognitive appraisal includes threat perception (i.e., assessing whether work activities are disturbing and harmful to people’s personal well-being), and challenge perception (i.e., assessing whether work activities are stimulating and exciting); and secondary cognitive appraisal includes coping potential (i.e., determining if the person’s personal resources are enough to handle the demands of the work), and control perception (i.e., evaluating whether the decision-making process in the work activity depends on personal control) [2,21,22]. Thus, the mediating role of cognitive appraisal in the relationship between stressful events and their ultimate outcomes shows that how an individual evaluates a stressful situation substantially affects the resulting consequences. The model also identifies different levels of responses that are implicitly involved in a stressful situation (e.g., psychological, emotional, physiological, and behavioral). The interactive process of appraisal and response describes an interactive process where the first level of cognitive appraisal influences responses, which in turn can lead to a second level of cognitive appraisal, suggesting a dynamic and continuous feedback loop. Finally, the model considers the outcomes of the stressful event and the adaptation process [2,22]. Also important, the interactive model of human adaptation to stress aligns with theoretical approaches dedicated to comprehending the academic stress of students, which also recognize the need to study the systemic conditions of academic stressors, the coping process to deal with stress, and the set of psychological, emotional, physiological, and behavioral responses of students [12,26]. Thus, in this study, stress was framed by transactional and interactive models that conceive this phenomenon as the result of the interaction between the individual and the environment, meaning that an event is considered stressful if the person appraises it as important and potentially harmful [21,22]. The same can be said for academic stress, resulting from a systemic adaptive process derived from academic demands (input), that can destabilize human functioning (unbalance indicators), requiring adaptive efforts from the person to restore the systemic balance (output) [26].

Considering this understanding of academic stress, in higher education, stress has become a significant concern, driven by two primary factors: increasing academic demands and expectations from the global job market. Students must not only learn academic content but also develop soft skills, gain practical experience, and build a strong personal brand. This dual pressure creates a situation in which academic performance is viewed as a clear indicator of future job prospects, resulting in increased emotional and mental stress. Recent research suggests that this pressure manifests in various ways, including task overload and anxiety about future employment, creating a cycle in which academic stress and job expectations reinforce each other. This indicates that academic stress is a multifaceted issue with substantial impacts on educational outcomes in higher education, particularly on academic performance [1]. Therefore, research on students’ academic stress underscores the importance of comprehending the adaptation process [26]. However, it has primarily focused on examining the relationship between academic stressors and students’ mental health, intending to mitigate adverse effects, enhance coping skills, and minimize the impact on academic performance and overall well-being [13,17]. Nevertheless, despite that recognition, no specific measures have been proposed to screen and evaluate the entire adaptation process, including its outcomes in higher education students. So, given the importance of screening and assessing stress experiences among higher education students, including their cognitive appraisal processes, coping resources, and their effects on human functioning and adaptation, this study advances the existing literature in two significant ways.

First, it proposes a battery of instruments that help us to understand university students’ adaptation to stress. Building on the transactional model [27] and the interactive model of human adaptation to stress [22,25], we propose a set of tools that assess the main stages of the stress adaptation process in higher education students. Based on the previous information about the model, we suggest a measure for each theoretical factor: to understand the antecedent factors of the model (i.e., sources of stress), the Stress Questionnaire for Students (SQS); for the cognitive appraisal processes, the Primary and Secondary Cognitive Appraisal Scale (PSCAS) and the Reduced Coping Inventory (Coping-R), that can also be considered a response to primary and secondary cognitive appraisals; and, for the outcome of event adaptation, the Academic Achievement Expectations (AAE). It should be noted that we use Coping-R as a measure of the interface between cognitive appraisal and stress responses, because the instrument assesses detailed coping responses, including those initiated immediately following exposure to a stressor and the consequent (primary and secondary) cognitive appraisals.

Second, the measures proposed in this study can further enhance theoretical understanding of the processes underlying human adaptation to stress as a continuous phenomenon in daily life settings, particularly in higher education settings. A key area for improvement in the current literature is the development and application of specific conceptual frameworks to explain people’s experiences of stress, how they assess and cope with it, and how these factors influence their functioning and adaptation to life situations, thereby supporting intervention.

Our study brings novelty to the current literature by testing, for the first time, the validity of a battery of four instruments with students (SQS, PSCAS, Coping-R, and AAE). Specifically, the SQS evaluates academic stressors related to academic performance (i.e., academic motivation, learning process, academic overload, and students’ concerns about their future and the financial resources necessary to fulfill their responsibilities as students). The main advantage of the SQS is that it integrates a broad set of stressors, evaluating equal dimensions of other instruments [8,28], but also evaluating dimensions less present in other instruments, such as, for example, concerns about the future and financial resources, reinforcing their value attributed by qualitative studies [9,29]. As for the PCAS, the main contribution of this instrument is that it allows for the evaluation of both primary and secondary cognitive appraisals, which is quite novel since most measures restrict evaluation to primary cognitive appraisal, namely the dimensions of threat and challenge [30,31]. Additionally, in our study, we will test for the first time the factor structure of the instrument in a student population. Regarding coping strategies, the main advantage of the Coping-R is that it provides a very structured version of coping dimensions: problem-solving (active coping), social support (emotional support), and emotion regulation, with the addition of whether coping is more active (humour) or passive (denial). Coping-R’s structure is quite unique because some other instruments seem to prefer spreading the evaluated coping dimensions extensively [32], eventually “losing” connection to the theoretical background of coping, besides the fact that it is difficult to stabilize their factor structures [33]. Finally, for academic achievement expectations (AAE), the main advantage of the instrument is that it provides a very short measure of students’ expectations to succeed, which is quite unique in this area. It is also the first to be tested in terms of validity in this study.

**Figure 1:**
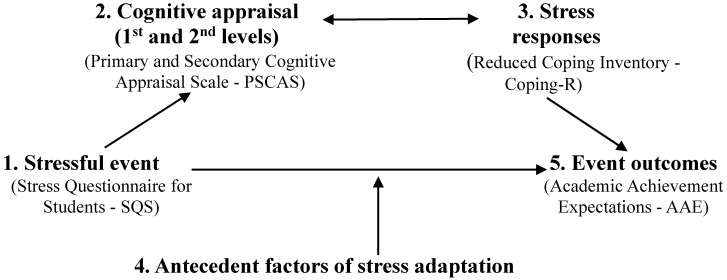
Interactive Model of Human Adaptation to Stress: Measures Used in our Study.

Considering all these factors, the main goal of this paper is to validate a set of tools that help us to understand how university students adapt to stress by specifically considering the theoretical framework of the interactive model of human adaptation to stress, as illustrated in Figure 1. Accordantly, this study has two specific goals: (a) to validate the psychometric properties of a battery of instruments that measure the constructs of the academic stress adaptation model (e.g., sources of stress, cognitive appraisal, coping, and academic achievement expectations); and (b) to develop and validate brief versions of a battery of instruments that assess the core constructs of the academic stress adaptation model (i.e., the same measures of the previous goal). All in all, our study adds to the current literature by testing and proposing a set of instruments that can be useful to analyze theoretically how students adapt to academic stress.

## 2. Method

### 2.1. Participants

This study included a convenience sample of 931 university students from three educational institutions in northern Portugal who voluntarily completed an online questionnaire. The mean age was 20.77 years (*SD* = 4.28), with a range of 17 to 54 years. The majority of participants were female (*n* = 689, 74%), 234 (25%) were male, and 8 (1%) preferred not to disclose their gender. Most participants were single (*n* = 893, 96%). In the total sample, 381 (41%) participants were from a public institution and 550 (59%) from a private institution, attending the courses of psychology (*n* = 356, 38%), health sciences (*n* = 229, 25%), and social sciences (*n* = 346, 37%). For most participants (*n* = 793, 85%), it was their first course option. Regarding the course attendance stage, 368 (39%) were in the first year of graduation, 154 (17%) were in the second year, 239 (26%) were in the third year, 50 (5%) were in the final stage of their graduation (fourth and fifth years), and 120 (13%) were in the first and second year of their master degree.

### 2.2. Instruments

#### 2.2.1. Sociodemographic Questionnaire

This questionnaire consists of a group of items concerning personal (e.g., age, gender, marital status) and academic (e.g., type of educational institution, course year, type of course) variables.

#### 2.2.2. Stress Questionnaire for Students (SQS)

The SQS assesses students’ perceptions of seven potential sources of stress they may experience in their academic activity. The instrument takes into account aspects that can trigger stress in students and the teaching process, as reinforced in the literature [8,9,34]. The full version includes 28 items that seek to assess structural dimensions of the student’s academic activity, organized into seven subscales of four items each: (a) academic performance: stress regarding academic results below the student’s expectations (e.g., “Having lower grades than I expected”; “Feeling like my grades aren’t what I want”); (b) academic evaluation: stress related to the evaluation moments that occur throughout the school year (e.g., “Taking tests/exams in subjects that I find difficult”; “Presenting assignments in class”); (c) motivation: stress concerning the low motivation in performing academic activities (e.g., “Feeling no motivation to study”; “Feeling that studying doesn’t motivate me much”); (d) learning: stress arising from the difficulty in keeping up with the teaching and learning process at school (e.g., “Not understanding some of the class content”; “Feeling “lost” when reading topics that I don’t understand well”); (e) work overload: stress related to the high load of academic activities and tasks (e.g., “Having a lot of schoolwork to do”; “Having a lot of school tasks to do/complete”); (f) future expectations: stress resulting from students not believing that their studies are valuable and stimulating (e.g., “Thinking that studying won’t bring me a better future”; “Not knowing what job I will get after finishing my school/degree/course”); and (g) financial problems: stress resulting from the student’s inability to continue studying due to finance and economic difficulties (e.g., “Thinking about the money I’m spending to study”; “Thinking about the financial burden it is to study”). This instrument includes an item in which the student must evaluate the overall level of academic stress they experience. The items are answered on a 5-point Likert scale (0 = *No stress at all*; 4 = *Very high stress*), and the score is computed by averaging the items in each dimension. Higher values indicate higher stress levels in each dimension.

#### 2.2.3. Primary and Secondary Cognitive Appraisal Scale (PSCAS)

The PSCAS was developed by Gomes and Teixeira [35] based on the transactional model of Lazarus [21,24,27], the interactive perspective of adaptation to stress [22], and the model of tension and pressure at work [36], seeking to reach a measure of an individual’s primary and secondary cognitive appraisal. The full version of the instrument [35] consists of 15 items organized into a total of five scales of three items each: (a) importance perception: evaluates the significance and relevance given to the situation (e.g., “My student activity … Is somewhat important to me”; “My student activity … Matters somewhat to me”); (b) threat perception: evaluates the disruptive and harmful potential of the situation (e.g., “My student activity… Is somewhat disturbing to me”; “My student activity… Is somewhat threatening to me”); (c) challenge perception: evaluates the stimulating and excitement potential of the situation (e.g., “My student activity… Is somewhat stimulating to me”; “My student activity… Is somewhat exciting to me”); (d) coping perception: assesses the personal resources to cope with the situation’s demands (e.g., “To what extent do you think you are able to deal and solve the demands of your student activity?”); and, (e) control perception: evaluates the perceived decision power over the situation (e.g., “To what extent do you feel that what happens in your student activity depends on you and your abilities?”). Items are answered on a 7-point Likert-type scale [(0 = It is not at all important to me; 6 = *It is very important to me*, for the importance of academic activities) regarding a specific situation (i.e., exam season)], and the score is computed by averaging the items in each dimension. Higher values indicate higher levels of cognitive appraisal in each dimension. Instructions to fulfil the instrument were adapted to academic activity, meaning that participants answered the questions thinking about their academic activity.

#### 2.2.4. Reduced Coping Inventory (Coping-R)

The reduced version of the Coping Inventory (Coping-R) is based on the work carried out for the COPE instrument by Carver, Scheier, and Weintraub [37] and Carver and Scheier [32]. The main difference proposed by Gomes is the reduction in the number of coping factors assessed, since it is not always possible to obtain positive data with the original factor structure [33]. Thus, this version proposes assessing strategies centered on problem-solving, emotion regulation, and social support. Specifically, the instrument assesses students’ coping strategies to deal with stress in their academic activities. This full version consists of 16 items answered on a 5-point Likert scale (1 = *Never used it*; 5 = *Used it often*), organized into four domains of coping, of four items each: (a) active coping: direct actions to solve the problem, considered a problem-solving strategy (e.g., “I try hard to solve the stressful situation”; “I do, “step by step”, what needs to be done to solve the stressful situation”); (b) humour: jokes or anecdotes intended to remove negative charge from the problem, considered a more active emotion regulation strategy (e.g., “I “joke” about the fact that the stressful situation is happening”; “I make fun about the stress I’m feeling”); (c) denial: thoughts aimed at denying the occurrence of the problem or its potentially negative consequences, considered a more passive emotion regulation strategy (e.g., “I act as if the stressful situation is not happening”; “I refuse to believe that the stressful situation is happening”); and (d) emotional support: talking and sharing the problem with other people to get emotional help, considered a social support focus strategy (e.g., “I talk to other people about how I’m feeling”; “I seek emotional support and understanding from others”). The score is computed by averaging the items in each dimension, and higher values indicate a higher level of coping resources in each dimension. Participants answered the questionnaire thinking about how they coped with the academic stress they felt at that moment.

#### 2.2.5. Academic Achievement Expectations (AAE)

The AAE assesses students’ perceptions of their academic achievement. The instrument consists of 5 items, each representing statements about achievement in a given area of academic activity (e.g., “I will successfully complete the academic year”; “I will be able to understand the subjects/topics in my classes”). The items are answered on a 5-point Likert scale (1 = *Definitely not*; 5 = *Definitely yes*), and the total score is computed by averaging all the items. Higher values indicate higher expectations of academic achievement.

### 2.3. Procedure

First, we submitted the study protocol to the Ethics Committee for Research at one of the authors’ institutions, which approved the study (CEICSH 034/2019). The data collection was conducted through an online questionnaire using the Qualtrics tool (Qualtrics), which included an informed consent form detailing the study’s goals, the voluntary nature of participation, and the anonymous nature of the data collected. During the collection period, the participating institutions publicized the protocol via electronic media, notifying the students of the link to the questionnaire. In this way, only students enrolled in the selected courses for our study were contacted, and only those who initially agreed to participate in the study were contacted by our research team. After this previous institutional contact, our research team made the electronic link available to the students. We collected the data at four different moments: in the middle of the first semester and at the end of the second semester of the 2021/22 and 2022/23 school years.

### 2.4. Data Analysis

The IBM SPSS (version 29.0) program was used for data analysis. The data analysis process involved four key steps.

In the first step, we performed an initial analysis to check the data for normality and multicollinearity assumptions, as well as to identify outliers and missing data. Thus, we started by screening the data for normality and multicollinearity assumptions, outliers, and missing data, following established criteria in the literature [38]. We used Kline’s [39] criteria of skewness ≤ |3| and kurtosis ≤ |10| to check for violations of normality assumptions. Additionally, we checked for multicollinearity by observing the VIF coefficients, considering established criteria in the literature [40].

In the second step, we randomly divided the total sample into two groups (the calibration sample and the validation sample), as suggested by Maroco [41]. We employed a calibration and validation strategy, where the first sample was used to test the original structure of the instruments and to calibrate and develop the short versions, and the second sample was used to validate the final short versions and to assess the model’s replicability. Then, we checked whether the sample sizes were adequate for the psychometric validity analysis using both exploratory factor analysis (EFA) and confirmatory factor analysis (CFA) [40,41].

In the third step, using the calibration sample, we conducted EFA on all original versions of the instruments as an initial step to assess their construct validity. Next, to evaluate the validity further and to identify the best final and short version for each instrument, we carried out CFA on all versions and tested for reliability and convergent and discriminant validity. Specifically, we performed EFA as a preliminary strategy to assess the instruments’ construct validity and to check for their theoretical dimensionality. Although with a previously solid theory-driven and a good fit for the CFA models, for all instruments, we follow Schmitt et al. [42] guidelines, recommending that it is wise to precede or follow up a CFA model with an EFA to understand the factor structure better when refining previously developed instruments and to test the factor structure’s generalizability [42]. For the EFA, we used the calibration sample. Therefore, regarding construct validity, the items for each scale were subjected to Principal Component Factor Analysis without pre-defining the number of factors, rather than using other common factorial methods, with a Direct Oblique Rotation (Oblimin) and eigenvalue ≥ 1 (considered the Kaiser criterion, along with Cattell’s standards for the scree plot graphical display and the parallel analysis to ascertain the number of factors to retain). The Kaiser-Meyer-Olkin Measure of Sampling Adequacy (KMO ≥ 0.6) and Bartlett’s Test of Sphericity (*p* < 0.05) enabled us to assess the suitability of the sample for conducting the factorial analysis. The sample adequacy of each item was measured using the anti-image matrix (MSA > 0.70; *r* < 0.09) [40,43]. The criteria for item retention were communalities values above 0.40 (ideally above 0.60), factor loadings above 0.50, few or no cross-loading items, while accepting the structure if it explained at least 50% of the total variance, and at least three items per factor [43,44]. Regarding the CFA, we performed structural equation modeling (SEM) to test the factor structure of all instruments by performing CFA in the calibration sample through the IBM SPSS AMOS (version 29.0; SPSS Inc., Chicago, IL., USA). In the first step to assess the instruments’ construct validity, we tested the structural models using maximum likelihood (ML) estimation methods. To estimate the model fit quality and to determine the extent to which the theoretical models replicate the correlational structure of the observed variables, we used the following fit indices: the chi-square goodness-of-fit statistics (χ^2^ test), and the absolute index χ^2^/*df*, the comparative fit index (CFI; ≥0.95 for good, ≥0.90 for acceptable), the Tucker–Lewis index (TLI; ≥0.95 for good, ≥0.90 for acceptable); the Steiger–Lind root mean square error of approximation (RMSEA; ≤0.06 for good, ≤0.08 for acceptable) with its 90% confidence interval (CI), and the standardized root mean square residual (SRMR; ≤0.06 for good, ≤0.08 for acceptable) [45] and the Akaike Information Criterion (AIC) [46]. To complement these indicators, we analyzed parameter estimates of the measurement models (e.g., factor loadings, cross-loadings, and correlations between factors) [45]. Additionally, when comparing models, we computed the difference in the chi-square statistic (∆χ^2^ test) [42] in addition to those indicators. We followed the most acceptable criteria described in the literature for the cutoff measures [39]. We tested the reliability of the instruments in the second step of assessing construct validity. For this purpose, we used the regression weights to evaluate the reliability of each item (for all items *λ* ≥ 0.50, *R*^2^ ≥ 0.25 for acceptable), the Cronbach’s alpha measure of internal consistency (α ≥ 0.70 for acceptable), and the composite reliability measure (CR) (CR ≥ 0.70 for acceptable) to evaluate construct reliability [40,47]. Also, in this third step, we tested the instruments’ convergent and discriminant validity by following the recommendations of Fornell and Larcker [48]. In particular, we assessed convergent validity through the average variance extracted (AVE) and the CR, verifying if the variance explained by the construct was greater than the measurement error and greater than the cross-loadings (i.e., AVE ≥ 0.50 and AVE < CR for acceptable) [47]. Additionally, we assessed discriminant validity by analyzing whether the AVE was higher than the maximum shared squared variance (MSV) or the average shared squared variance (ASV). Accordingly, the shared variance (R^2^_i__j_) between two subscales of the same instrument should not exceed either the individual AVE values of the subscales (AVE_i_ and AVE_j_) or their average, thereby supporting discriminant validity (i.e., AVE_i_ and AVE_j_ ≥ R^2^_i__j_) [47].

Finally, we used the validation sample for cross-validation analysis. Specifically, we assessed the construct validity of the final short version of the instrument through CFA and by testing reliability, convergent, and discriminant validity, using the same analysis criteria described above in the third step.

## 3. Results

### 3.1. Scales Development Procedure

The scale development followed the same procedure for the SQS, the PSCAS, the Coping-R, and the AAE instruments. All instruments had specific theoretical models as a basis (see instrument description), converging in the interactive perspective of adaptation to stress [22]. Also important, the development of the instruments followed the ‘best practice’ recommendations for psychological instruments development. Specifically, we provide in our study indications about the constructs evaluated by each instrument, how items were generated, data about items and construct validity and reliability, and cross-validation analysis, all of which represent main tasks of development of psychological instruments [49,50,51].

The development of each instrument followed literature analysis of each construct. Thus, the team started by establishing the theoretical construct evaluated in each instrument. The SQS intends to evaluate a set of academic stressors that can change the usual patterns of human functioning in students, and that can be more or less positive for the students, depending on the way they evaluate each one of them. Some literature and similar instruments were analyzed to establish the specific dimensions of the SQS, with particular attention being given to academic stressors related to academic performance, academic motivation, the learning process, academic overload, future expectations, and financial problems, among other stressful factors for students [8,9,29,52].

The PCAS intends to evaluate the cognitive process of analyzing whether a certain demand represents a threat/harm or a challenge/benefit to the individual’s well-being due to the feelings of having, or not having, the necessary resources and control to meet the demands of the stressor. The PCAS derives directly from transactional [21,24] and interactive [2,22] models, and further studies using this instrument in other populations [35]. It is now being tested in university students.

The Coping-R intends to evaluate a set of coping strategies that a person can use to deal with the demands of a certain life event, and is influenced by primary and secondary cognitive appraisals, which can, in turn, influence the stress responses. The development of this instrument followed an analysis of similar instruments of coping [33,37], trying to stabilize the number of coping factors into problem-solving (active coping), social support (emotional support), and emotion regulation, in a more active way (humour) and in a more passive way (denial). This reduction captures the main domains of coping, namely task-focused coping (i.e., which involves dealing with the problem causing distress), emotion-focused coping (i.e., which involves dealing with the negative emotions of the problem causing distress) [27,53], adding also the coping focused on emotional support (i.e., which involves seeking social and emotional support or support from others) [54].

The AAE intends to evaluate the academic achievement expectations that students have about the possibility of achieving success in their academic activity. Academic achievement represents a main criterion in school effectiveness studies [55] and is frequently used as a school effectiveness indicator by stakeholders in education [56]. In fact, there are indications that students who feel positive about themselves are likely to be intrinsically motivated and tend to be academically competent [57], meaning that there is a positive relationship between academic self-concept and academic achievement as a student [58]. Thus, the AAE tries to evaluate educational expectations related to future achievements [59,60,61], as is the case with the expectation to understand the subjects of classes, the expectation to complete the tasks required in classes, the expectation to fulfil personal goals, the expectation to be successful as a student, and the expectation to reach a future of success.

Regarding item generation, the expert team responsible for this task consisted of six PhD researchers (five from the psychology field and one from the sports science field) and two Master of Psychology (MPsych) researchers. The selection of the expert team conciliated distinct factors: (a) experience/knowledge in educational university contexts; (b) experience in psychological instruments development and data analysis; and (c) experience/knowledge in occupational stress. Thus, all PhD researchers had at least five years of experience; one of the MPsych researchers had one year of experience, and the other had 15 years of working experience in the educational field, in topics related to human adaptation to stress. The first stage consisted of item generation for each instrument (viz., one team was involved in this task), and the second stage consisted of the final item selection for each instrument (viz., another team was involved in this task). The ultimate goal was to develop two final versions of the instruments, each with a balance of items per factor, ideally not exceeding four, as recommended by Bollen [62].

In stage one, the criteria followed by the first team were: (1) generating items with language clarity that resembled the original theoretical factors; (2) providing a final version with six items per factor to be reduced in the second stage. Each member completed the first task independently (i.e., four PhD researchers assumed this task, with two assigned to each instrument). The second task involved a team meeting to discuss the item generation. Following this procedure, teams first analyzed the proposed items (i.e., teams switched tasks and analyzed the instrument of the other team). Then, they analyzed the items again with the four researchers present.

In stage two, the second team followed three criteria: (1) analyzing the language clarity (i.e., words and sentences of items), (2) analyzing the theoretical conformity (i.e., appropriateness of the item to measure the original factor), and (3) providing instruments with four or three items per factor. The first task was completed in small groups to discuss opinions, and a final group debate occurred to select the final items. Ultimately, we proposed the first version of all instruments: 28 items for SQS (four items per factor), 16 items for the Coping-R (four items per factor), 15 items for the PSCAS (three items per factor), and five items for the AAE. After this phase, we collected data to test the psychometric properties of the instruments.

### 3.2. Preliminary Analysis

First, regarding the total sample (*N* = 931), we screened the data for normality and multicollinearity assumptions, outliers, and missing data. We did not find missing data nor observed influential outliers based on the median absolute deviation. Additionally, no violations of normality assumptions were observed in item distribution, and all VIF coefficients regarding multicollinearity assumptions were below five, indicating no issues. Second, we randomly divided the total sample (*N* = 931) into two groups: (1) the calibration sample, corresponding to 60% of cases (*n* = 561), and (2) the validation sample, corresponding to 40% of cases (*n* = 370). The sample sizes were adequate for the psychometric validity analysis: exploratory factor analysis (EFA) and confirmatory factor analysis (CFA) [40,41]. Both samples remained representative of the initial study population. We repeated the same screening procedure for normality and multicollinearity assumptions for each group created, and no issues were detected [39,40].

### 3.3. Exploratory Factor Analysis

#### 3.3.1. EFA for the SQS

We conducted the EFA for the SQS (28-item version) (KMO = 0.930, Bartlett’s Test = 10,439.218, *df* = 378, *p* < 0.001), which revealed a factorial structure of six dimensions (rather than the expected seven) while explaining 70.193% of the total variance. All items loaded higher than 0.50 on a single factor, except for item 18 from the academic evaluation (0.465), which also exhibited the lowest communality value (0.354). Item 23 from the academic evaluation dimension presented a weight load on a different factor, and the remaining items (i.e., items 9, 18, and 27) are weight-loaded together, as with those from the learning dimension (i.e., items 6, 15, 20, 28). These last showed a higher weight load (ranging from 0.743 to 0.821). We found communality values below 0.60 for item 23 (0.545, academic evaluation), item 2 (0.544, future expectations), and item 21 (0.579, motivation), with the remaining items being higher. An “EFA” with a pre-definition of seven factors revealed that items from the academic evaluation presented weight loads in distinct factors (i.e., items 9 and 27 in the same factor as learning, and the remaining 18 and 23 in two other factors), suggesting that the academic evaluation and learning items probably assess the same dimension. In this regard, despite the EFA revealing inconsistencies in how the 28 items loaded across the seven factors, we continued with CFA in AMOS, drawing on earlier publications showing adequate fit indices for this factorial structure (see references earlier).

#### 3.3.2. EFA for the PSCAS

The EFA for the PSCAS (15-item version) (KMO = 0.860, Bartlett’s Test = 4280.745; *df* = 105; *p* < 0.001) showed a factorial structure of four dimensions and not the expected five, explaining 69.42% of the total variance. All items loaded higher than 0.50 on a single factor, with items from importance and challenge loading on the same factor. The items’ communality values were adequate (i.e., >0.40): item 9 presented the lowest communality value (0.446); the remaining communality was higher than 0.60 (viz., [0.602; 0.828]). In an “EFA” with a pre-definition of five factors, the items presented the expected distribution (three items per factor). However, item 9, regarding challenge perception, presented cross-loading in its factor and importance perception. Despite some issues identified in the EFA (viz., item 9), we conducted the CFA in AMOS, guided by previous studies that reported an adequate model fit for the five-factor structure [35].

#### 3.3.3. EFA for the Coping-R

The EFA for the Coping-R (16-item version) (KMO = 0.845, Bartlett’s Test = 5658.275, *df* = 120, *p* < 0.001) showed a factorial structure of four dimensions as expected, explaining 74% of the total variance. All items loaded higher than 0.70 on a single factor, and the items’ communality values were adequate (i.e., >0.40): item 1 presented the lowest communality value (0.554); the remaining items’ communality was higher than 0.60 (viz., [0.617; 0.879]). In the face of these results and guided by previous studies that reported an adequate model fit for the four-factor structure, we conducted the CFA in AMOS.

#### 3.3.4. EFA for the AAE

The EFA for the AAE (5-item version) (KMO = 0.822, Bartlett’s Test = 444.917, *df* = 10, *p* < 0.001) revealed a unifactorial structure solution, explaining 60.41% of the total variance. All items loaded above 0.70 on a single factor, and the items’ communality values were satisfactory (i.e., >0.40): items 2 and 1 had the lowest communality values (0.548 and 0.569), while the remaining items’ communality was greater than 0.60 (viz., [0.605; 0.678]). Considering these results and guided by previous studies that reported an adequate model fit for the one-factor structure, we conducted the CFA in AMOS.

### 3.4. Confirmatory Factor Analysis (Latent Structure)

#### 3.4.1. Confirmatory Factor Analysis for the SQS

Regarding the latent structure of the SQS (28-item version), the results from the CFA showed that the seven-factor model (with four items per factor) fitted the data well. Specifically, we obtained the following tests of significance and goodness-of-fit measures: χ^2^(329) = 1039.489, *p* < 0.001, χ^2^/*df* = 3.160, CFI = 0.931, PCFI = 0.810, TLI = 0.920, RMSEA = 0.062 (90% C.I. [0.058; 0.066], *p*close < 0.001), SRMR = 0.0429, AIC = 1193.489, BIC = 1526.877. All items presented acceptable factor weight loadings (λ ≥ 0.50) and item-total correlations (>0.40). So, to proceed with a reduced version of 21 items, we removed one item per dimension based on the lower factor weight loading. Accordingly, we removed the following items: 2 (λ = 0.621; future expectations), 4 (λ = 0.654; academic performance), 6 (λ = 0.796; learning), 8 (λ = 0.656; financial problems), 18 (λ = 0.507; academic evaluation), 21 (λ = 0.658; motivation), and 25 (λ = 0.818; work overload). The results for the CFA for the SQS (21-item version) showed that the seven-factor model (three items per factor) fitted the data well, as indicated by the following measures: χ^2^(168) = 471.057, *p* < 0.001, χ^2^/*df* = 2.804, TLI = 0.951, CFI = 0.961, PCFI = 0.768, RMSEA = 0.057 (90% C.I. [0.051; 0.063], *p*close = 0.033), SRMR = 0.0354, AIC = 597.057, BIC = 869.830. The difference between the 28-item version and the 21-item version [∆χ^2^(161) = 568,432, *p* < 0.001] shows that the latter presents a better adjustment. Table 1 shows the reliability measures for SQS versions, and Table 2 displays the inter-construct correlations.

Results in Table 1 show some validity reservations in the 28-item and 21-item versions of the SQS. The 28-item version presents convergent validity questions: an AVE = 0.473 for academic evaluation lower than the cutoff of 0.5; and discriminant validity concerns: an MSV = 0.839 for academic evaluation and an MSV = 0.839 for learning, greater than their AVE, and an SR_AVE = 0.688 for academic evaluation and an SR_AVE = 0.838 for learning lower than the inter-construct correlations between academic evaluation and learning (r = 0.916). Results for the 21-item version present the same discriminant validity concerns regarding academic evaluation and learning (i.e., MSV > AVE), with a consistently high correlation between the two factors (*r* = 0.913) and superior to SR_AVE values, suggesting that they evaluate the same construct.

Given these results, we tested a six-factor model for the 21-item version, joining all items from academic evaluation and learning in the same factor. Based on the lower factor weight load and cross-loadings, we removed the three items regarding academic evaluation [viz., item 23 (λ = 0.633), item 9 (λ = 0.719), and item 27 (λ = 0.758)] to achieve a final version with three items per factor. The CFA for the final Version of the SQS with 18 items revealed that the six-factor model (three items per factor) fitted the data well, as indicated by the following measures: χ^2^(120) = 283.374, *p* < 0.001, χ^2^/*df* = 2.361, TLI = 0.968, CFI = 0.975, PCFI = 0.765; RMSEA = 0.040 (90% C.I. [0.042; 0.057], *p*close = 0.549), SRMR = 0.0324, AIC = 385.374, BIC = 606.190. All items presented adequate factorial weight loads (viz., λ ≥ 0.50; [0.667; 0.909]), as presented in Table 3. The difference between the 18-item version and both the 28-item version [∆χ^2^(209) = 756,115, *p* < 0.001] and the 21-item version [∆χ^2^(48) = 187,683, *p* < 0.001] is statistically significant, showing that the 18-item version is better. The SQS 18-item version exhibits better psychometric properties in terms of construct validity and reliability (see Table 1 and Table 2).

#### 3.4.2. Confirmatory Factor Analysis for the PSCAS

Results for the CFA of the PSCAS (15-item version) indicated that the five-factor model (three items per factor) fitted the data well. Namely, we obtained the following tests of significance and goodness-of-fit measures: χ^2^(80) = 270.648; *p* < 0.001; χ^2^/*df* = 3.383, TLI = 0.941, CFI = 0.955, PCFI = 0.727, RMSEA = 0.065 (90% C.I. [0.057; 0.074], *p*close = 0.002), SRMR = 0.0585, AIC = 350.648, BIC = 523.837. All items had adequate factor weight loadings (viz., *λ* ≥ 0.50), ranging from *λ =* 0.505 to *λ =* 0.883, except for item 9 (challenge perception), which also presented the lowest factor weight loading (*λ =* 0.379). So, to proceed with a reduced version of 10 items for intervention purposes, we removed one item per dimension based on the lower factor weight loading. Specifically, we removed the following items: 2 (*λ* = 0.842; importance perception), 5 (*λ* = 0.728; threat perception), 9 (*λ* = 0.379; challenge perception), 12 (*λ* = 0.722; coping perception), and 13 (*λ* = 0.505; control perception). The results for the CFA for the PSCAS (10-item version) showed that the five-factor model (two items per factor) fitted the data well, as indicated by the following measures: χ^2^(25) = 75.469, *p* < 0.001; χ^2^/*df* = 3.019, TLI = 0.966, CFI = 0.981, PCFI = 0.545, RMSEA = 0.06 (90% C.I. [0.045; 0.076], ***p***close = 0.133), SRMR = 0.0323, AIC = 135.469, BIC = 265.36. All items presented adequate factorial weight loads (viz., *λ* ≥ 0.50; [0.667; 0.927]) (as presented in Table 4). The difference between the 15-item version and the 10-item version [∆χ^2^(55) = 195.179, *p* < 0.001] shows that the latter presents a better adjustment. Table 5 shows the reliability and the convergent and discriminant validity measures for the PSCAS versions, and Table 6 displays the inter-construct correlations.

The results presented in Table 5 demonstrate adequate convergent and discriminant validity for both the 15-item and 10-item versions of the PSCAS for students. However, regarding the PSCAS 15-item convergent validity, the “control perception” factor yielded an AVE of 0.480, which falls slightly below the recommended threshold of 0.50 [48]. Despite this marginal deviation, the standardized factor loadings for this factor are acceptable (viz, 0.505, 0.853, 0.684), and both the composite reliability (CR = 0.729) and Cronbach’s alpha (α = 0.719) show satisfactory internal consistency. Furthermore, the correlations between perceived control and the other factors ranged from *r* = 0.333 to *r* = 0.522, all statistically significant at *p* < 0.001 (Table 5), suggesting that the constructs remain conceptually distinct. Therefore, although the AVE is marginally below the conventional cutoff, the overall pattern of results supports the adequacy of this factor, suggesting a borderline yet acceptable level of convergent validity, particularly given that this was the only factor with such a deviation.

#### 3.4.3. Confirmatory Factor Analysis for the Coping-R

Results of the CFA for the Coping-R (16-item version) showed that the four-factor model (four items per factor) fit the data well. Specifically, we obtained the following significance tests and goodness-of-fit measures: χ^2^(98) = 344.645, *p* < 0.001, χ^2^/*df* = 3.517, TLI = 0.946, CFI = 0.956, PCFI = 0.781, RMSEA = 0.067 (90% C.I. [0.059; 0.075], *p*close < 0.001), SRMR = 0.0503, AIC = 420.645, BIC = 585.174. All items showed adequate factor loadings (viz., *λ* ≥ 0.50), ranging from *λ* = 0.593 to *λ* = 0.936. To continue with a reduced version of 12 items, we removed one item per dimension based on the lower factor loading. Specifically, we removed the following items: 1 (*λ* = 0.602; active coping), 2 (*λ* = 0.852; humour), 6 (*λ* = 0.817; emotional support), and 15 (*λ* = 0.593; denial). The CFA for the Coping-R (12-item Version) showed that the four-factor model (three items per factor) fitted the data well, as shown by the following measures: χ^2^(48) = 174.167, *p* < 0.001; χ^2^/*df* = 3.628, TLI = 0.956, CFI = 0.968, PCFI = 0.704, RMSEA = 0.069 (90% C.I. [0.058; 0.080], ***p***close = 0.003), SRMR = 0.0418, AIC = 234.167, BIC = 364.059. All items presented adequate factorial weight loads (viz., *λ* ≥ 0.50; [0.636; 0.942]) (Table 7). The difference between the 16-item version and the 12-item version [∆χ^2^(50) = 170.478, *p* < 0.001] shows that the latter presents a better adjustment. Table 8 shows the reliability and the convergent and discriminant validity measures for the Coping-R versions, and Table 9 displays the inter-construct correlations.

Table 8 shows that both the 16-item and 12-item versions of the Coping-R exhibit adequate convergent and discriminant validity on a global scale. However, the convergent validity of the Coping-R 16-item version indicates that the “Active Coping” factor has an AVE of 0.483, slightly below the ideal threshold of 0.50 [48]. Despite this minor shortfall, the standardized factor loadings for this factor remain acceptable (viz., 0.760, 0.636, 0.763), and both the composite reliability (CR = 0.787) and Cronbach’s alpha (α = 0.784) demonstrate suitable internal consistency. Additionally, the correlations between Active Coping and the other factors ranged from *r* = 0.099 to *r* = 0.528, all statistically significant at *p* < 0.05 (Table 9), showing that the constructs remain conceptually distinct. Thus, while the AVE is just slightly below the traditional cutoff, the overall results affirm the adequacy of this factor, indicating a borderline yet acceptable level of convergent validity, particularly since this is the only factor showing such a deviation. Furthermore, the data do not indicate any issues with the convergent or discriminant validity of the 12-item Version of Coping-R, confirming it as the preferred version of the instrument.

#### 3.4.4. Confirmatory Factor Analysis for the AAE

Results of the CFA for the AAE (5-item version) showed that the one-factor model fit the data. Specifically, we obtained the following significance tests and goodness-of-fit measures: χ^2^(5) = 23.218, *p* < 0.001, χ^2^/*df* = 4.644, TLI = 0.917, CFI = 0.959, PCFI = 0.479, RMSEA = 0.122 (90% C.I. [0.075; 0.174], *p*close = 0.008), SRMR = 0.040, AIC = 43.218, BIC = 78.271. All items showed adequate factor loadings (viz., *λ* ≥ 0.50), ranging from *λ* = 0.638 to *λ* = 0.797. To achieve a better adjustment, we tested the same factorial structure by adding the error correlation suggested—item 2 correlated with item 3 (*r* = 0.273, *p* < 0.001) and item 1 (*r* = 0.208, *p* = 0.004). results identify a better adjustment: χ^2^(3) = 3.51, *p* = 0.319, χ^2^/*df* = 1.17, TLI = 0.996, CFI = 0.999, PCFI = 0.30, RMSEA = 0.026 (90% C.I. [0.0001; 0.114], *p*close = 0.559), SRMR = 0.0141, AIC = 27.51, BIC = 69.574. All items showed adequate factor loadings (viz., λ ≥ 0.50), ranging from λ = 0.552 to λ = 0.834. On reliability, Cronbach’s alpha measure of 0.834 showed an acceptable and good internal consistency of the items (α ≥ 0.70). The item-total correlation values were higher than 0.40 and ranged from 0.592 to 0.691, showing that all items make a significant contribution to the construct.

### 3.5. Cross-Validation Analysis

We continued with a CFA in the validation sample to cross-validate the factorial structure of all instruments and to attest to their psychometric properties in terms of construct validity and reliability.

#### 3.5.1. Cross-Validation Analysis for the SQS

Regarding the SQS-18 item version, the six-factor model (three items per factor) fitted the data well, as indicated by the following measures: χ^2^(120) = 221.749; *p* < 0.001; χ^2^/*df* = 1.848, TLI = 0.970, CFI = 0.977, PCFI = 0.766, RMSEA = 0.048 (90% C.I. [0.038; 0.058], *p*close = 0.623), SRMR = 0.0385, AIC = 323.749, BIC = 523.338. For all items, regression weights to evaluate the reliability of each item were acceptable (*λ* ≥ 0.50), ranging from 0.654 to 0.937. Concerning convergent validity, the AVE was higher than 0.50, ranging from 0.617 (motivation) to 0.811 (financial problems), and the AVE was inferior to the CR. Regarding discriminant validity, the MSV was inferior to the AVE, and the SR_AVE was greater than the inter-construct correlations, which ranged from 0.234 to 0.756. We did not find reliability concerns: the Cronbach’s alpha measure was acceptable (α ≥ 0.70), ranging from 0.827 (motivation) to 0.927 (financial problems), and an adequate CR (CR ≥ 0.70), ranging from 0.829 (motivation) to 0.928 (financial problems).

#### 3.5.2. Cross-Validation Analysis for the PSCAS

Analyzing the PSCAS 10-item version, the CFA showed that the five-factor model (two items per factor) fit the data well. Specifically, we obtained the following tests of significance and goodness-of-fit measures: χ^2^(25) = 46.053, *p* = 0.006; χ^2^/*df* = 1.842, TLI = 0.978, CFI = 0.988, PCFI = 0.549, RMSEA = 0.048 (90% C.I. [0.025; 0.069], *p*close = 538), SRMR = 0.0311, AIC = 106.053, BIC = 223.458. Additionally, the regression weights used to evaluate the reliability of each item were above the threshold (*λ* ≥ 0.50), ranging from 0.708 to 0.916. Concerning convergent validity, the AVE was higher than the cutoff (0.50), with values ranging from 0.542 (control perception) to 0.780 (importance perception), and the CR exceeded the AVE. Regarding discriminant validity, the MSV was lower than the AVE, and the SR_AVE was greater than the inter-construct correlations, which ranged from 0.233 to 0.592. We did not observe any reliability concerns: the Cronbach’s alpha measure was acceptable (α ≥ 0.70), ranging from 0.702 (control perception) to 0.875 (coping perception), and the CR was suitable (CR ≥ 0.70), ranging from 0.703 (control perception) to 0.876 (importance perception).

#### 3.5.3. Cross-Validation Analysis for the Coping-R

Regarding the Coping-R 12-item version, the four-factor model (three items per factor) fit the data well, as indicated by the following measures: χ^2^(48) = 102.49, *p* < 0.001, χ^2^/*df* = 2.135, TLI = 0.969, CFI = 0.978, PCFI = 0.711, RMSEA = 0.055 (90% C.I. [0.041; 0.07], *p*close = 0.258), SRMR = 0.0410, AIC = 162.49, BIC = 279.895. Regression weights assessing each item’s reliability were adequate (λ ≥ 0.50), ranging from 0.657 to 0.926. Concerning convergent validity, the AVE was greater than 0.50, ranging from 0.533 (Active Coping) to 0.777 (Humour), while the AVE was lower than the CR. For discriminant validity, the MSV was less than the AVE, and the SR_AVE was greater than the inter-construct correlations, which ranged from 0.065 to 0.616. We found no reliability issues: the Cronbach’s alpha measure was appropriate (α ≥ 0.70), ranging from 0.773 (Active Coping) to 0.911 (Humour), alongside an adequate CR (CR ≥ 0.70), ranging from 0.774 (Active Coping) to 0.913 (Humour).

#### 3.5.4. Cross-Validation Analysis for the AAE

**Figure 2:**
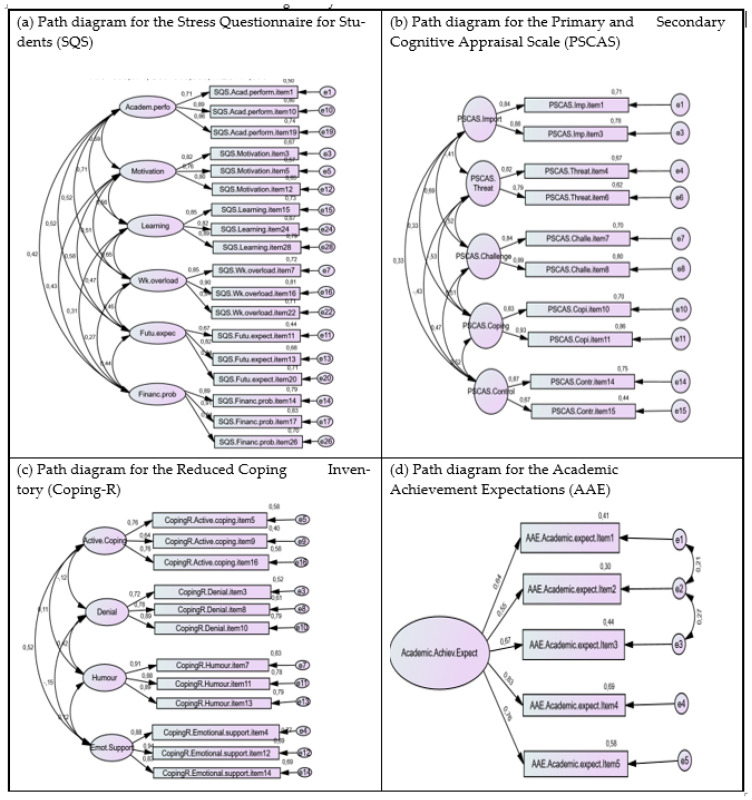
Path diagrams of the models.

Regarding the results for the AAE CFA, the one-factor model fits the data, as indicated by the following measures: χ^2^(3) = 4.889, *p* = 0.018, χ^2^/*df* = 1.63, TLI = 0.971, CFI = 0.991, PCFI = 0.297, RMSEA = 0.068 (90% C.I. [0.001; 0.172], *p*close = 0.307), SRMR = 0.025, AIC = 28.889, BIC = 64.103. The item’s factor loadings ranged from λ = 0.494 to λ = 0.752. In terms of reliability, Cronbach’s alpha measure of 0.796 indicated an acceptable level of internal consistency for the items (α ≥ 0.70). The item-total correlation values were above 0.40 (ranging from 0.416 to 0.700), demonstrating that all items significantly contributed to the construct. Figure 2 includes the path diagrams for the short versions of the instruments.

All in all, our study proposes four instruments that can be used to evaluate the process of adaptation to academic stress in students by assuming the theoretical background of the interactive model of human adaptation to stress.

## 4. Discussion

This study tested a set of tools that can facilitate the understanding of how higher education students adapt to stress by analyzing the psychometric validity of the original forms of the instruments and by developing shorter versions with enhanced psychometric properties.

Based on Lazarus’ transactional model [27] and the interactive model of human adaptation to stress [22], we propose a battery of instruments to evaluate all stages of the stress adaptation process in higher education students. Specifically, we proposed the Stress Questionnaire for Students (SQS) to assess students’ sources of stress; the Primary and Secondary Cognitive Appraisal Scale [35] to assess students’ cognitive appraisal processes; the Reduced Coping Inventory (Coping-R) to measure students’ coping responses that can directly derive from cognitive appraisal of the stressor; and the Academic Achievement Expectations (AAE) to evaluate students’ perception of academic performance as an adaptation outcome.

Generally, all instruments seem to be valid and reliable when assessed using the standard ‘best practice’ guidelines for developing psychological tools [49]. Starting with the Stress Questionnaire for Students, the results show a final and reduced version with 18 items and a six-factor structure as the best solution, supporting the instrument’s validity in terms of construct validity, reliability, convergent validity, and discriminant validity [47,48], when compared with the original form (i.e., the 28-item version). In fact, our data indicated that statistical parameters related to model fit (e.g., ∆χ^2^ test) were better in the shorter version than in the longer version. This instrument focuses on the structural aspects of the student’s academic activity, addressing potential sources of stress related to situations where academic results fall short of students’ expectations (i.e., Academic performance); reduced motivation to accomplish academic tasks and demands (i.e., Motivation); the students’ difficulty in accompanying the teaching and learning processes within the educational context (i.e., Learning); the heavy load of academic activities and tasks (i.e., Work overload); the students’ skepticism regarding the value and engagement of their studies (i.e., Future expectations); and the student’s inability to continue studying due to financial and economic hardships (i.e., Financial problems).

The SQS categorization aligns with the literature, indicating that academic stress among university students originates from multiple sources. It highlights factors such as academic workload—numerous heavy assignments, tight deadlines, and frequent exams, which create significant pressure [63,64], allied to the pressure to maintain high academic standards, can result in test anxiety and burnout [63]. Studies also indicate that the lack of clarity in feedback from instructors can exacerbate feelings of inadequacy and confusion [63]. Additionally, difficulties in understanding course material and unexpected challenges in research proposals contribute to stress [63,65]. High expectations from family and society create additional pressure, leading to feelings of impostor syndrome and a fear of failure [64]. As a result, students often struggle with motivation, particularly when facing overwhelming academic demands [17,66]. Additional significant sources of stress among students include financial constraints and obligations [67], which significantly impact students’ mental health as they manage tuition fees, living costs, and potential debt [64,66]. This can detract from academic focus and performance [65] and even lead to dropping out of education [18]. Therefore, understanding these factors is essential for creating effective support systems. Also important, the SQS aligns with similar instruments dedicated to evaluate the stress of university students, having as major advantage the possibility of evaluating distinct dimensions proposed separately by other instruments, as is the case of the academic activity of students [8,28], the coping and psychophysiological responses [68,69], and the conditions surrounding the students activity, as for example the familiar and economic conditions [70,71,72,73]. In this way, the SQS 18-item version can be utilized in research settings and practical applications, particularly in psychological assessments and intervention environments, allowing for the evaluation of the first variable in the interactive model of human adaptation to stress [22].

Regarding the Primary and Secondary Cognitive Appraisal Scale, the original 15-item version with a five-factor structure showed adequate construct validity and reliability in university students, replicating the results obtained in previous studies [5], including a validation study with health professionals [35]. Additionally, the results of the shorter version with a 10-item, five-factor structure version are a better option, supporting the instrument’s validity in terms of construct validity, reliability, and convergent and discriminant validity [47,48], compared to the 15-item version. In fact, our data indicated that statistical parameters related to model fit (e.g., ∆χ^2^ test) were better in the shorter version than in the longer version. The PSCAS aims to assess a student’s primary and secondary cognitive appraisal regarding their academic activities. The instrument assesses the importance students assign to the academic situation (i.e., perception of importance), its potential to be disruptive or damaging (i.e., threat perception), and its exciting possibilities (i.e., challenge perception)—all of which relate to students’ primary cognitive appraisal processes [2,27]. It also includes students’ perception of their personal resources for coping with academic demands (i.e., coping perception) and their perceived decision-making power over the situation (i.e., control perception)—which pertain to students’ secondary cognitive appraisal processes [2,27]. Cognitive appraisals play a crucial role in how university students perceive and respond to academic stressors, particularly in high-pressure academic contexts. The primary appraisal involves an initial evaluation of the situation’s relevance and potential consequences, which significantly influences students’ emotional experiences [74]. This initial assessment determines whether a situation is perceived as stressful or manageable, setting the stage for subsequent emotional responses [5]. Following this, the secondary appraisal process evaluates the students’ coping abilities and available resources, guiding their behavioral responses to the stressor [75]. Together, these appraisals form the cognitive appraisal model, emphasizing the importance of understanding students’ perceptions to create effective interventions that enhance their resilience and emotional well-being [76]. By addressing both primary and secondary appraisals, educators can develop targeted strategies that help students overcome academic challenges more effectively. Thus, the PSCAS enables the assessment of the second variable in the interactive model of human adaptation to stress [25,27]. The PCAS aligns with similar instruments dedicated to evaluate the cognitive appraisal processes, having as major advantage the possibility of evaluating both the primary and secondary cognitive appraisals, there existing other instruments more dedicated to evaluate these two processes separately, specially primary cognitive appraisal [30,77].

Concerning the Reduced Coping Inventory (Coping-R), the results show a final and reduced version with 12 items and a four-factor structure as the best solution, supporting the instrument’s validity in terms of construct validity, reliability, and convergent and discriminant validity [47,48], when compared with the original form (i.e., the 16-item version). In fact, our data indicated that statistical parameters related to model fit (e.g., ∆χ^2^ test) were better in the shorter version than in the longer version. This instrument assesses students’ coping strategies related to problem-solving, emotion regulation, and social support. It includes students’ direct actions to resolve problems (i.e., active coping); discussing and sharing the problem with others for emotional support (i.e., emotional support); using jokes or anecdotes to reduce the negativity of the issue (i.e., humour); and students’ thoughts aimed at denying the occurrence of the problem or its potential adverse effects (i.e., denial). In this respect, the high levels of academic stress that university students often face underscore the importance of identifying and promoting effective coping strategies. The literature consistently categorizes coping efforts into adaptive and maladaptive strategies, each with distinct implications for student well-being and academic performance. Adaptive coping strategies, particularly those focused on problem-solving, such as time management, planning, and seeking academic support, have been shown to mitigate stress and enhance academic performance [63,78]. Emotion-focused strategies, including mindfulness practices, social support, and setting realistic goals, also contribute positively to students’ psychological well-being and emotional regulation [78,79]. Moreover, coping flexibility (i.e., the ability to use different strategies, such as positive reappraisal and help-seeking) has been linked to higher self-efficacy and resilience, both of which serve as protective factors in the academic environment [79]. In contrast, maladaptive coping strategies like avoidance behaviors, excessive gaming, substance use, and social withdrawal are often associated with increased stress and worse outcomes [78,80]. These coping patterns not only fail to solve academic problems but also harm mental health, raise dropout chances, and, in severe cases, lead to suicidal thoughts [80,81]. While adaptive coping is vital for managing academic pressures, the widespread use of maladaptive strategies among students underscores an urgent need for institutional intervention. Universities must develop comprehensive support systems that foster healthier coping techniques, enhancing both academic success and overall mental health. Thus, the 12-item Coping-R version can be used in both research and practical settings, such as in psychological assessments and interventions, to evaluate the second variable in the interactive model of human adaptation to stress, while emphasizing students’ specific coping strategies. In synthesis, the Coping-R allows for the evaluation of the main dimensions of coping (i.e., problem-solving, emotion regulation, and social support) [27,53] with a stable factor structure of four dimensions, something that does not occur in other similar instruments [33].

Lastly, regarding Academic Achievement Expectations (AAE), the results indicate that the one-factor structure supports the instrument’s validity in terms of construct validity and reliability [40,47]. This instrument evaluates students’ perceptions of their academic success. It can be used in research or intervention settings as a measure of their functioning, reflecting the outcome of their adaptation process and their experience with academic stress. As noted in the literature, the experience of stress can influence a student’s perception of academic performance. High levels of academic stress are consistently linked to lower academic performance, with students reporting decreases in grades that correlate with higher stress levels [4]. Also important, there are indications that students’ expected future achievement relates to their self-efficacy beliefs [59,61], meaning that there is a relationship between students’ academic skills and the development of expectations about future achievement [60].

Some limitations need to be addressed. Although the study relied on a convenience sample, two random groups with comparable characteristics were formed, allowing for the cross-validation of the proposed instruments. This methodological approach enhances the robustness of the findings, despite possible limitations in generalizability. Future research should aim to include a broader range of academic courses to support and further expand these findings, extending the sample to students of other academic areas (e.g., Sciences, Legal Sciences, Arts, Technology, Engineering and Architecture, and Mathematics). Also important, all instruments can also be tested (and adapted) for other students, besides the ones in university contexts, being quite possible that the stress questionnaire needs to include other stress factors for students in elementary contexts. Finally, future studies can also test the convergent validity of the instruments by comparing them with other similar measures.

Regarding practical implications, these findings highlight the crucial need for universities to implement evidence-based stress management strategies that promote both academic performance and students’ overall well-being [82]. These approaches can be theoretically guided by transactional and interactive approaches of human adaptation to stress, given the existing data showing that the dimensions of the interactive model of human adaptation to stress [22] proved to be important in samples of university teachers [83], health professionals [84], and even athletes [85].

## 5. Conclusions

The present study offers a set of empirically grounded tools for assessing stress in higher education contexts, enabling a comprehensive understanding of the adaptive process. This includes identifying potential stressors, analyzing students’ cognitive appraisal of these factors, evaluating their perceived control and coping potential, and examining the specific coping strategies employed. By integrating these dimensions, the approach also facilitates the identification of students’ expectations regarding academic success, thereby informing targeted interventions and institutional support mechanisms. In summary, the instruments seem to be appropriate for studying the adaptation process of students, and may represent suitable measures for future research. The final versions of the questionnaires are provided in Appendix A of this study.

## Figures and Tables

**Table 1:** Reliability and Convergent and Discriminant Validity for the SQS.

SQS Versions	Alpha	CR	MaxR(H)	AVE	MSV	SR_AVE
28-item Version						
1. Academic performance	0.861	0.865	0.952	0.619	0.601	0.787
2. Academic evaluation	0.768	0.778	0.960	0.473	0.839	0.688
3. Motivation	0.841	0.845	0.968	0.578	0.406	0.760
4. Learning	0.904	0.904	0.975	0.703	0.839	0.838
5. Work overload	0.910	0.911	0.982	0.719	0.587	0.848
6. Future expectations	0.822	0.826	0.978	0.545	0.406	0.738
7. Financial problems	0.890	0.896	0.920	0.687	0.218	0.829
21-item Version						
1. Academic performance	0.859	0.864	0.949	0.681	0.604	0.826
2. Academic evaluation	0.780	0.783	0.957	0.547	0.834	0.740
3. Motivation	0.836	0.836	0.965	0.631	0.343	0.794
4. Learning	0.889	0.888	0.973	0.726	0.834	0.852
5. Work overload	0.897	0.898	0.981	0.745	0.529	0.863
6. Future expectations	0.817	0.824	0.977	0.612	0.336	0.782
7. Financial problems	0.909	0.910	0.915	0.771	0.195	0.878
18-item Version						
1. Academic performance	0.859	0.864	0.979	0.681	0.511	0.825
2. Motivation	0.835	0.836	0.941	0.631	0.343	0.794
3. Learning	0.888	0.889	0.961	0.727	0.511	0.853
4. Work overload	0.896	0.898	0.975	0.745	0.428	0.863
5. Future expectations	0.814	0.824	0.968	0.612	0.336	0.782
6. Financial problems	0.909	0.910	0.915	0.771	0.195	0.878

Note. Alpha, Cronbach’s alpha; AVE = Average Variance Extracted; CR = Composite Reliability; MaxR (H) = Maximum Reliability; MSV = Maximum Shared Squared Variance; SR_AVE = Square root of AVE.

**Table 2:** Inter-Construct Correlation for the SQS.

**SQS: 28-Item Version**	**1. AP**	**2. AE**	**3. M**	**4. L**	**5. WO**	**6. FE**	**7. FP**
1. Academic performance	1						
2. Academic evaluation	0.775 ***	1					
3. Motivation	0.596 ***	0.597 ***	1				
4. Learning	0.698 ***	0.916 ***	0.591 ***	1			
5. Work overload	0.548 ***	0.766 ***	0.539 ***	0.689 ***	1		
6. Future expectations	0.543 ***	0.528 ***	0.637 ***	0.482 ***	0.474 ***	1	
7. Financial problems	0.409 ***	0.376 ***	0.423 ***	0.302 ***	0.287 ***	0.467 ***	1
**SQS: 21-Item Version**	**1. AP**	**2. AE**	**3. M**	**4. L**	**5. WO**	**6. FE**	**7. FP**
1. Academic performance	1						
2. Academic evaluation	0.777 ***	1					
3. Motivation	0.586 ***	0.560 ***	1				
4. Learning	0.714 ***	0.913 ***	0.556 ***	1			
5. Work overload	0.518 ***	0.727 ***	0.515 ***	0.653 ***	1		
6. Future expectations	0.516 ***	0.492 ***	0.580 ***	0.473 ***	0.448 ***	1	
7. Financial problems	0.417 ***	0.367 ***	0.434 ***	0.307 ***	0.266 ***	0.442 ***	1
**SQS: 18-Item Version**	**1. AP**	**2. AE**	**3. M**	**4. L**	**5. WO**	**6. FE**	**7. FP**
1. Academic performance	1						
3. Motivation	0.584 ***		1				
4. Learning	0.756 ***		0.594 ***	1			
5. Work overload	0.424 ***		0.470 ***	0.557 ***	1		
6. Future expectations	0.501 ***		0.506 ***	0.477 ***	0.363 ***	1	
7. Financial problems	0.373 ***		0.320 ***	0.383 ***	0.234 ***	0.430 ***	1

Note. *** *p* < 0.001.

**Table 3:** Subscales, Items, and Factor Weight Loads for the SQS 18-Item Version.

Subscales (QSE: 18-Item Version)	Items | [Likert Scale: 0, 1, 2, 3, 4]
Academic performance (3 items)	1 (*λ* = 0.708), 10 (*λ* = 0.893), 19 (*λ* = 0.863) | Total = 0–4
Motivation (3 items)	3 (*λ* = 0.820), 5 (*λ* = 0.758), 12 (*λ* = 0.803) | Total = 0–4
Learning (3 items)	15 (*λ* = 0.853), 24 (*λ* = 0.816), 28 (*λ* = 0.888) | Total = 0–4
Work overload (3 items)	7 (*λ* = 0.847), 16 (*λ* = 0.899), 22 (*λ* = 0.843) | Total = 0–4
Future expectations (3 items)	11 (*λ* = 0.667), 13 (*λ* = 0.825), 20 (*λ* = 0.843) | Total = 0–4
Financial problems (3 items)	14 (*λ* = 0.887), 17 (*λ* = 0.909), 26 (*λ* = 0.837) | Total = 0–4
	Total = 18 items

**Table 4:** Subscales, Items, and Factor Weight Loads for PSCAS 10-Item Version.

Subscales (PSCAS: 10-Item Version)	Items | [Likert Scale: 0, 1, 2, 3, 4, 5, 6]
1. Importance Perception (2 items)	1 (*λ* = 0.842), 3 (*λ* = 0.882) | Total = 0–6
2. Challenge Perception (2 items)	7 (*λ* = 0.839), 8 (*λ* = 0.893) | Total = 0–6
3. Threat Perception (2 items)	4 (*λ* = 0.821), 6 (*λ* = 0.788) | Total = 0–6
4. Coping perception (2 items)	10 (*λ* = 0.835), 11 (*λ* = 0.927) | Total = 0–6
5. Control Perception (2 items)	14 (*λ* = 0.866), 15 (*λ* = 0.667) | Total = 0–6
	Total = 10 items

**Table 5:** Reliability, Convergent, and Discriminant Validity for PSCAS.

PSCAS Versions	Alpha	CR	MaxR(H)	AVE	MSV	SR_AVE
15-item Version						
1. Importance Perception	0.893	0.894	0.895	0.737	0.484	0.858
2. Challenge Perception	0.727	0.765	0.963	0.545	0.484	0.738
3. Threat Perception	0.824	0.827	0.969	0.615	0.295	0.784
4. Coping Perception	0.861	0.866	0.942	0.685	0.295	0.828
5. Control Perception	0.719	0.729	0.953	0.480	0.274	0.695
10-item Version						
1. Importance Perception	0.851	0.853	0.856	0.743	0.480	0.862
2. Challenge Perception	0.856	0.857	0.961	0.751	0.480	0.866
3. Threat Perception	0.784	0.786	0.966	0.647	0.277	0.805
4. Coping Perception	0.871	0.875	0.935	0.778	0.277	0.882
5. Control Perception	0.728	0.745	0.948	0.597	0.272	0.773

Note. Alpha, Cronbach’s alpha; AVE = Average Variance Extracted; CR = Composite Reliability; MaxR (H) = Maximum Reliability; MSV = Maximum Shared Squared Variance; SR_AVE = Square root of AVE.

**Table 6:** Inter-Construct Correlation for the PSCAS.

**PSCAS: 15-Item Version**	**1. Importance**	**2. Challenge**	**3. Threat**	**4. Coping**	**5. Control**
1. Importance Perception	1				
2. Challenge Perception	0.696 ***	1			
3. Threat Perception	−0.359 ***	−0.468 ***	1		
4. Coping Perception	0.351 ***	0.497 ***	−0.543 ***	1	
5. Control Perception	0.357 ***	0.469 ***	−0.412 ***	0.523 ***	1
**PSCAS: 10-Item Version**	**1. Importance**	**2. Challenge**	**3. Threat**	**4. Coping**	**5. Control**
1. Importance Perception	1				
2. Challenge Perception	0.693 ***	1			
3. Threat Perception	−0.413 ***	−0.521 ***	1		
4. Coping Perception	0.332 ***	0.512 ***	−0.526 ***	1	
5. Control Perception	0.333 ***	0.469 ***	−0.435 ***	0.522 ***	1

Note. *** *p* < 0.001.

**Table 7:** Subscales, Items, and Factor Weight Loads for Coping-R 12-Item Version.

Subscales (Coping-R: 12-Item Version)	Items | [Likert Scale: 1, 2, 3, 4, 5]
Active Coping (3 items)	5 (*λ* = 0.760), 9 (*λ* = 0.636), 16 (*λ* = 0.763) | Total = 1–5
Emotional Support (3 items)	4 (*λ* = 0.877), 12 (*λ* = 0.942), 14 (*λ* = 0.832) | Total = 1–5
Denial (3 items)	3 (*λ* = 0.718), 8 (*λ* = 0.780), 10 (*λ* = 0.891) | Total = 1–5
Humor (3 items)	7 (*λ* = 0.912), 11 (*λ* = 0.882), 13 (*λ* = 0.887) | Total = 1–5
	Total = 12 items

**Table 8:** Reliability, Convergent, and Discriminant Validity for Coping-R.

Coping-R Versions	Alpha	CR	MaxR(H)	AVE	MSV	SR_AVE
16-item version						
Active Coping	0.784	0.787	0.803	0.483	0.279	0.695
Emotional Support	0.923	0.924	0.972	0.754	0.279	0.868
Denial	0.830	0.838	0.976	0.569	0.172	0.754
Humor	0.932	0.934	0.951	0.779	0.172	0.883
12-item version						
Active Coping	0.761	0.765	0.775	0.521	0.275	0.722
Emotional Support	0.913	0.915	0.967	0.783	0.275	0.885
Denial	0.831	0.841	0.973	0.639	0.180	0.800
Humor	0.921	0.923	0.940	0.799	0.180	0.894

Note. Alpha, Cronbach’s alpha; AVE = Average Variance Extracted; CR = Composite Reliability; MaxR (H) = Maximum Reliability; MSV = Maximum Shared Squared Variance; SR_AVE = Square root of AVE.

**Table 9:** Correlation Between Constructs for Coping-R.

**Coping-R: 16-Item Version**	**1. Active Coping**	**2. Emotional Support**	**3. Denial**	**4. Humor**
1. Active Coping	1			
2. Emotional Support	0.528 ***	1		
3. Denial	−0.112 *	−0.125 **	1	
4. Humor	0.099 *	0.107 *	0.415 ***	1
**Coping-R: 12-Item Version**	**1. Active Coping**	**2. Emotional Support**	**3. Denial**	**4. Humor**
1. Active Coping	1			
2. Emotional Support	0.524 ***	1		
3. Denial	−0.121 *	−0.150 **	1	
4. Humor	0.107 *	0.119 *	0.424 ***	1

Note. *** *p* < 0.001. ** *p* < 0.01. * *p* < 0.05.

## Data Availability

The data is not publicly available and complies with data protection regulations.

## Appendix A

**SQS—General version (Students)**
  **Part 1**

On the following scale, indicate the number that best describes the level of stress that you usually feel in your student activity.

In general, my student activity causes me …

No stress at all	Not much stress	Some stress	High stress	Very high stress
0	1	2	3	4

**Part 2**

Below are presented some potential sources of stress for students. Please indicate the number that best indicates the level of stress generated by each potential source of stress in **your student activity** (0 = *No stress at all*, 2 = *Some stress*; 4 = *Very high stress*).

**Table:**

**For Each of the Following Situations, Please Indicate the Level of Stress You Feel in Your STUDENT ACTIVITY…**	**No Stress at All**	**Not Much Stress**	**Some Stress**	**High Stress**	**Very High Stress**
1. Having lower grades than I expected	0	1	2	3	4
2. Feeling no motivation to study	0	1	2	3	4
3. Feeling that studies don’t motivate me much	0	1	2	3	4
4. Having a lot of schoolwork to do	0	1	2	3	4
5. Feeling like my grades aren’t what I want	0	1	2	3	4
6. Thinking that studying won’t bring me a better future	0	1	2	3	4
7. Feeling lower desire to do schoolwork	0	1	2	3	4
8. Not knowing what job I will get after finishing school/degree/course	0	1	2	3	4
9. Thinking about the money I’m spending to study	0	1	2	3	4
10. Not understanding some of the class content	0	1	2	3	4
11. Having a lot of school tasks to do/complete	0	1	2	3	4
12. Thinking about the financial burden it is to study	0	1	2	3	4
13. Not having the academic success I would like	0	1	2	3	4
14. Fearing what might happen to me after I finish my course/degrees	0	1	2	3	4
15. Having too much schoolwork for the available time	0	1	2	3	4
16. Feeling “lost” when reading topics that I don’t understand well	0	1	2	3	4
17. Feeling that studying is a very expensive activity	0	1	2	3	4
18. Realizing that I don’t understand some subjects/topics	0	1	2	3	4
SQS Codification					
1. Academic performance: 1, 5, 13 2. Motivation: 2, 3, 7 3. Learning: 10, 16, 18 4. Work overload: 4, 11, 15 5. Future expectations: 6, 8, 14 6. Financial problems: 9, 12, 17					

Note: The items were first used in the Portuguese language and then they were translated to English using the back translation method, but future studies may analyse the appropriateness of the translation.

**PSCAS (Students)**

Below are some questions related to your student activity. Please mark with a circle the number that best indicates how you **usually feel** in your student activity.

**Table:**

	**Is Not** **Important to Me**		**Is Somewhat Important to Me**			**Is Very Important** **To Me**			
1. My student activity…	0	1	2	3	4	5	6		
	Does not matter to me		Matters somewhat to me			Matters a lot to me			
2. My student activity…	0	1	2	3	4	5	6		
	Means nothing to me		Means something to me			Means a lot to me			
3. My student activity…	0	1	2	3	4	5	6		
	Is not disturbing to me		Is somewhat disturbing to me			Is very disturbing to me			
4. My student activity…	0	1	2	3	4	5	6		
	Is not threatening to me		Is somewhat threatening to me			Is very threatening to me			
5. My student activity…	0	1	2	3	4	5	6		
	Is not negative to me		Is somewhat negative			Is very negative to me			
6. My student activity…	0	1	2	3	4	5	6		
	Is not stimulating to me		Is somewhat stimulating to me			Is very stimulant to me			
7. My student activity…	0	1	2	3	4	5	6		
	Is not exciting to me		Is somewhat exciting to me			Is very exciting to me			
8. My student activity…	0	1	2	3	4	5	6		
	Is not challenging to me		Is somewhat challenging to me			Is very challenging to me			
9. My student activity…	0	1	2	3	4	5	6		
		Not able at all				Somewhat able		Very able	
10. To what extent do you think you are able to deal and solve the demands of your student activity?			0	1	2	3	4	5	6
		Not prepared at all				Somewhat prepared		Very well prepared	
11. To what extent do you think you are prepared to deal and solve the demands of your student activity?			0	1	2	3	4	5	6
		Not able at all				Somewhat able		Very able	
12. To what extent do you think you are able to have a good performance in your student activity?			0	1	2	3	4	5	6
		Does not depends on me				Depends somewhat on me		Depends on me	
13. To what extent do you feel that what happens in your student activity depends on you and your abilities?			0	1	2	3	4	5	6
		Low control				Some control		High control	
14. To what extent do you feel that you control what should be done in your student activity?			0	1	2	3	4	5	6
		Low power of decision				Some power of decision		High power of decision	
15. To what extent do you feel that you have the power to decide what to do in your student activity?			0	1	2	3	4	5	6
CAS Codification|Complete Version			PCAS Codification|Brief Version						
A. Primary cognitive appraisal 1. Importance perception: 1, 2, 3 2. Threat perception: 4, 5, 6 3. Challenge perception: 7, 8, 9 B. Secondary cognitive appraisal 4. Coping perception: 10, 11, 12 5. Control perception: 13, 14, 15			A. Primary cognitive appraisal 1. Importance perception: 1, 3 2. Threat perception: 4, 5 3. Challenge perception: 7, 8 B. Secondary cognitive appraisal 4. Coping perception: 10, 12 5. Control perception: 14, 15						

Note: the items were first used in Portuguese language and then they were translated to English using back translation method, but future studies may analyse the appropriateness of the translation.

Coping-R|Students|General complete version

Try to think about the situations that generate the most levels of stress in your activity as a student. Then, indicate for each statement below whether you will use the strategy described to manage the stress you experience as a student.

Be aware that there are no right or wrong answers, but rather different ways to cope with problems and stressful situations.

**Table:**

**Usually, When Facing Stressful Situations as a Student…**	**I never Use It**	**I Use It a Little**	**I Use It Sometimes**	**I Use It Quite Often**	**I Always Use It**
1. I try hard to solve the stressful situation	1	2	3	4	5
2. I “joke” about the fact that the stressful situation is happening	1	2	3	4	5
3. I act as if the stressful situation is not happening	1	2	3	4	5
4. I talk to other people about how I’m feeling	1	2	3	4	5
5. I do, “step by step”, what needs to be done to solve the stressful situation	1	2	3	4	5
6. I seek emotional support and understanding from others	1	2	3	4	5
7. I make fun about the stress I’m feeling	1	2	3	4	5
8. I refuse to believe that the stressful situation is happening	1	2	3	4	5
9. I take direct action to solve the stressful situation	1	2	3	4	5
10. I pretend the stressful situation isn’t happening	1	2	3	4	5
11. I make jokes about the stressful situations	1	2	3	4	5
12. I talk to people who understand me and help me feel better	1	2	3	4	5
13. I “laugh” about the stressful situation	1	2	3	4	5
14. I seek support from people important to me to help me deal with how I feel	1	2	3	4	5
15. I tell myself “this isn’t happening”	1	2	3	4	5
16. I try different strategies/solutions to solve the stressful situation	1	2	3	4	5
Coping R Codification|Complete Version	Coping R Codification|Brief Version				
1. Active coping: 1, 5, 9, 16 2. Emotional support: 4, 6, 12, 14 3. Humour: 2, 7, 11, 13 4. Denial: 3, 8, 10, 15	1. Active coping: 5, 9, 16 2. Emotional support: 4, 12, 14 3. Humour: 7, 11, 13 4. Denial: 3, 8, 10				

Note: the items were first used in the Portuguese language and then they were translated to English using the back translation method, but future studies may analyse the appropriateness of the translation.

**AAE|Academic Achievement Expectations**

This questionnaire evaluates your opinion about your academic performance. Try to think about what is realistic and possible for you to achieve as a student this academic year. Please indicate your opinion on a scale of 1 (“Certainly not”) to 5 (“Certainly yes”) and take into consideration the fact that there are no right or wrong answers.

**Table:**

**This School/Academic Year…**	**Certainly Not**	**Almost Certainly Not**	**Maybe**	**Almost Certainly Yes**	**Certainly Yes**
1. I will successfully complete the academic year	1	2	3	4	5
2. I will be able to understand the subjects/topics in my classes	1	2	3	4	5
3. I will successfully complete the tasks required by my teachers	1	2	3	4	5
4. I will achieve the goals I established for this school/academic year	1	2	3	4	5
5. I will continue on my path to future success	1	2	3	4	5
AAE codification					
Academic Achievement Expectations: 1, 2, 3, 4, 5					

Note: the items were first used in Portuguese language and then they were translated to English using back translation method, but future studies may analyse the appropriateness of the translation.
